# Dynamics of social media behavior before and after SARS-CoV-2 infection

**DOI:** 10.3389/fpubh.2022.1069931

**Published:** 2023-02-23

**Authors:** Francesco Durazzi, François Pichard, Daniel Remondini, Marcel Salathé

**Affiliations:** ^1^Department of Physics and Astronomy (DIFA), University of Bologna, Bologna, Italy; ^2^Digital Epidemiology Lab, School of Life Sciences, School of Computer and Communication Sciences, Ecole Polytechnique Fédérale de Lausanne (EPFL), Global Health Institute, Geneva, Switzerland

**Keywords:** COVID-19, Twitter, deep learning, symptoms, Natural Language Processing (NLP)

## Abstract

**Introduction:**

Online social media have been both a field of research and a source of data for research since the beginning of the COVID-19 pandemic. In this study, we aimed to determine how and whether the content of tweets by Twitter users reporting SARS-CoV-2 infections changed over time.

**Methods:**

We built a regular expression to detect users reporting being infected, and we applied several Natural Language Processing methods to assess the emotions, topics, and self-reports of symptoms present in the timelines of the users.

**Results:**

Twelve thousand one hundred and twenty-one twitter users matched the regular expression and were considered in the study. We found that the proportions of health-related, symptom-containing, and emotionally non-neutral tweets increased after users had reported their SARS-CoV-2 infection on Twitter. Our results also show that the number of weeks accounting for the increased proportion of symptoms was consistent with the duration of the symptoms in clinically confirmed COVID-19 cases. Furthermore, we observed a high temporal correlation between self-reports of SARS-CoV-2 infection and officially reported cases of the disease in the largest English-speaking countries.

**Discussion:**

This study confirms that automated methods can be used to find digital users publicly sharing information about their health status on social media, and that the associated data analysis may supplement clinical assessments made in the early phases of the spread of emerging diseases. Such automated methods may prove particularly useful for newly emerging health conditions that are not rapidly captured in the traditional health systems, such as the long term sequalae of SARS-CoV-2 infections.

## 1. Introduction

Public health monitoring is in the midst of a technological shift enabled by the availability and pervasiveness of real-time and geo-localized data. Mining, harmonizing, and extracting information from heterogeneous big data sources is offering unprecedented opportunities in disease early warning and surveillance (1). Compared with information extracted from traditional public health channels, digital resources can reduce the timeframe of outbreak detection and improve our understanding of risk factors at the level of entire populations (2).

In particular, communicable diseases are currently drawing the most attention in studies of digital public health surveillance (3). In the context of the ongoing COVID-19 pandemic, multiple attempts have been made by scientists and public health institutions to address the challenge raised by the disease spread through new technologies (4). On the one hand, efforts have been made to provide practical solutions to the containment of infections through digital contact tracing (5) and innovative diagnostic and case management practices (6). On the other hand, digital traces can be processed to now-cast the number of infections and monitor the evolution of patients' symptoms or public reaction to the pandemic over time (4). Online social media are certainly one of the most fruitful sources of data, and even more so since the beginning of the pandemic that led to an increased use of these web platforms (14% more users on Twitter from January 2020 to April 2020) (7, 8). The real-time analysis of social media was developed across several themes, including surveillance of public attitudes, characterization of infodemics, assessing mental health, detecting or forecasting COVID-19 cases, and identifying the government interventions (9).

The heterogeneous nature of the available online data suggests that social and psychological phenomena beyond the strictly sanitary context can be observed and studied. Research has largely focused on large-scale social considerations, which revealed the increasing polarization of the COVID-19 debate (10) and the prevalence of negative emotions in messages posted on Twitter, particularly at the beginning of the outbreak (7, 11). Additionally, it was shown that the high number of tweets with negative sentiment was partly due to the high rate of (i) angry tweets in response to news from authorities and politics, and (ii) sad tweets in response to news about confirmed cases and deaths (12). Although such studies allow estimating the prevalence of mental health issues associated with the pandemic status of COVID-19 at the population level (7), there has been—to the best of our knowledge—no social media investigation of behavioral changes in individuals infected with SARS-CoV-2.

From a medical point of view, it has been highlighted that the number of self-reports of symptoms displayed a temporal correlation with the number of confirmed cases (13, 14). The frequency of symptoms reported on Twitter was shown to be in good agreement with the prevalence of symptoms following confirmed infections (15) and allowed to identify patterns revealing the long-term criticality of the post-acute sequelae of COVID-19 (16). However, in order to carry out these studies, time-consuming practices of manual annotation and curation had to be employed, which limited the amount of usable data.

Given the aforementioned gaps, we tried to leverage the full potential of social media data by using automated methods for the filtering of very large volumes of tweets as well as the characterization of a broad set of topics. We were thus able to investigate the content changes on Twitter for users who publicly shared that they had tested positive for COVID-19. Our hypothesis at the early stage of this work was that several features of the content posted by Twitter users had potentially changed after they reported their SARS-CoV-2 infection. These changes may concern not only health statuses, but also emotions, topics, external references (i.e., websites). Using various Natural Language Processing (NLP) techniques, we extracted heterogeneous information from the tweets, and characterized the users' timelines by designing a pre-post study to observe content changes after the infection self-reports (test-positive tweets from now on). We thus verified the time correlation between test-positive tweets and confirmed cases in the major English-speaking countries and performed statistical tests to assess differences between the pre-period and the post-period with respect to the test-positive date.

## 2. Materials and methods

### 2.1. Data

Using the Crowdbreaks platform (17), we collected a stream of COVID-related tweets through the filtered streaming endpoint of the Twitter API. This dataset of around 323M original tweets consists of all the English-language tweets (i) posted from 13/01/2020 to 19/09/2021 and (ii) mentioning COVID-19 related keywords (“wuhan,” “ncov,” “coronavirus,” “COVID,” “SARS-CoV-2”). Tweets that were selected were those satisfying the condition imposed by the following regular expression:

r'/b (?<!/“)(?:I|We) (?: have |/'ve |ve | just |) tested positive for (?:covid|corona|sars-cov-2)|/b (?<!/”) (?:my|our) (?:covid|corona|sars-cov-2) test is positive|/b (?<!/“) (?:found out|turns out|confirms|confirm) I (?:ve|/'ve| have| got| contracted) (?:covid|corona|sars-cov-2)'.

This regular expression was built *via* multiple steps of refinement, as we read random samples of matched tweets and modified the filter to avoid false positives. Our primary aim was not to retrieve all users reporting their infection on Twitter; rather, we were concerned with collecting a sample large enough to run sound statistical analyses (hence our willingness to accept a lower sensitivity when prioritizing a high specificity). Still, because of the rigidity of regular expressions, we may have missed a non-negligible portion of users reporting the positivity of their test to COVID-19. For each one of the users having at least one match with the regular expression, we downloaded the full publicly available Twitter timeline from 1/1/2020 to 30/09/2021 calling the Twitter API v2.0 (18) for Academic research through the Python's package Twarc (19). Due to Twitter limitations, we could only retrieve the last 3,000 tweets written by each user up to 30/09/2021 (day of the API call). Ethical approval for the use of the data was obtained from the EPFL Human Research Ethics Committee (054-2022).

### 2.2. Digital cohort selection

The common point between all users included in the dataset is that they reported testing positive to COVID-19 on Twitter. For each user, a pre-period (12 weeks) and a post-period (12 weeks) were defined with respect to the time of the so-called primary test-positive tweets, which marks the end of the pre-period and the start of the post-period. The tweets written by each user during this observation period were the main focus of our study and were used in all of the analyses. Some users reported testing positive to COVID-19 more than once, leading to a set of 268 secondary test-positive tweets. As our goal was to characterize changes in the users' Twitter timelines after their earliest SARS-CoV-2 infection online report, our analysis of self-reports of COVID-19 cases was based on the former set (primary test-positive tweets) only. Twitter accounts were further filtered according to the time of the primary test-positive tweets (25/03/2020 - 08/07/2021) and the number of tweets present in the pre- and the post-periods (at least 30 tweets per period posted over 5 weeks or more). This way, we were able to build a digital cohort of 12,121 users, who wrote a total of 5,932,306 tweets during the time of analysis. All subsequent analyses refer to this set of Twitter users.

### 2.3. Named Entity Recognition methods for symptoms and time expressions

Symptom mentions in tweets were extracted with MedCAT, which is a state-of-the-art Named Entity Recognition (NER) tool built to recognize and normalize clinical concepts in electronic health records (20). NER is an information extraction task carried out by a (statistical) model such that pre-defined categories are assigned as tags to the entities identified during the parsing of an unstructured text. MedCAT enabled to associate each tweet with a particular set of tags (no symptom, one symptom, or multiple symptoms encountered). However, after reading some of MedCAT's output, we realized that this model was limited due to its inability to recognize figurative uses of symptoms (e.g., “This situation hurts me,” “I am sick of this situation”), and we decided not to consider some symptom tags (“Pain,” “Sickness,” “Tired” and “Ache”) for the remainder of our analysis.

Since MedCAT was used beyond its original scope (processing of electronic health records) and applied to tweets, we compared the respective performances of MedCAT and a lexicon-based method developed for Twitter data (15). The authors of the latter study (Sarker et al.) were interested in extracting self-reports of COVID-19 symptoms from tweets and had manually reviewed the results of their model so as to obtain high levels of recall (15). By comparing the two methods we realized that MedCAT outperformed the lexicon-based approach over a set of manually annotated symptom-containing tweets, in particular considering the specificity (0.41 for MedCAT vs. 0.26 for the lexicon; *cf*. Supplementary material for details). Furthermore, “fatigue” and “tiredness” are disambiguated with MedCAT, which is not the case with the lexicon-based approach. As explained above, tags of tiredness were prone to being false positives, but this very problem did not occur with “fatigue.” Therefore, MedCAT enabled us to conduct analyses on self-reports of fatigue.

While hundreds of different symptom types may be recognized in our dataset, we decided to limit our analysis to a subset of symptoms pertaining to COVID-19 and Long COVID (i.e., post-acute sequelae of SARS-CoV-2 infection): fatigue, malaise, dyspnea, chest pain, fever, coughing, headache, sore throat, nausea, vomiting, dizziness, myalgia (16, 21, 22).

In order to tell apart actual self-reports of symptoms from generic chatter about symptoms, we used a fine-tuned deep learning model that aims to classify tweets with symptom mentions as either self-reports, non-personal reports, or news mentions (model with 3 classes) (23). Tweets with symptom mentions were retained for the following analysis if and only if their probability for being a self-report was larger than 0.9.

### 2.4. Emotion classification

A NER model was applied to determine which emotions were expressed by individual users as they posted content on Twitter. The model used (SpanEmo) (24) was a multilabel classifier with 11 possible labels including the eight primary (25) emotions (joy, trust, fear, surprise, sadness, disgust, anger, anticipation) and three dyadic emotions (optimism, pessimism, love). If none of these 11 labels was predicted the tweet was considered as neutral.

### 2.5. Topic classification

Topics present in the tweets were identified using the distilled version of the BART-Large-MNLI model (26). Nine custom topic categories were defined for this zero-shot learning classifier, namely: Business & Industry, Computers & Internet & Electronics, Education & Reference, Entertainment & Music, Health, Politics & Government & Law, Science & Mathematics, Society & Culture, Sport. The model returns the probability for a tweet to be associated with a particular topic. In order to use the aforementioned topics as categorical features, the indicator function was applied to the output probabilities (a particular topic is present in a tweet only if the estimated probability for that topic is above 0.5). If the indicator function was equal to 0 for all nine classes, the tweet was considered as an instance of a 10th category called “Other.”

### 2.6. Individual-level causal impact analyses on the volume of tweets

The following analysis was conducted for each user separately. First, user-specific tweets were split into two sets depending on their temporal position relative to the user's test-positive tweet (pre-intervention period or post-intervention period) and the timelines of all the users were aligned so that all the test-positive tweets happen to be on day 0. Then, numerical information was derived for every user by computing the weekly counts of tweets posted by individual accounts. Based on these user-specific time series, we were able to treat each user in the dataset separately and we tested whether the infection event (reflected by a given user's test-positive tweet) had a causal impact on the volume of tweets that were subsequently posted. To do so, we used the *CausalImpact* module of the Python package *tfcausalimpact* (27) (see Supplementary Figure 1). The statistical effect of the intervention (self-report of a given user's test-positive status) is inferred from the Bayesian one-sided tail-area probability of obtaining this effect by chance. The outcome of the model was one of the three following options depending on the effect and the magnitude of the *p*-value (statistical significance set at the 0.05 level): (i) the intervention (self-report of a given user's test-positive status) did *not* trigger a change in the user's posting volume; (ii) the intervention led to an *increased* activity; (iii) the intervention led to a *decreased* activity.

### 2.7. Paired tests for the change of proportions in labeled tweets

Using the same individual time series as for the previous analysis, we determined the average weekly number of tweets posted in the pre-period and the corresponding value in the post-period, obtaining a pair of pre-post counts for each user (12,121 pairs). Considering these individual data altogether, we applied a two-sided Wilcoxon signed-rank test in order to determine whether the rates in the pre-period and the post-period series were statistically different from each other. The results were computed with the R library *stats* (28). Pre-/post-comparisons of this type (i.e., Wilcoxon signed-rank test) were also conducted to statistically analyze the proportions of tweets associated with a particular class across all selected users. The possible classes correspond to the set of labels retrieved from the different models applied in this study (emotion/topic/URL/symptom classifiers). Wilcoxon signed-rank tests were applied with the false discovery rate controlled at the 0.05 level following the Benjamini-Hochberg *post-hoc* procedure.

### 2.8. Time duration of the increase in symptom reports posted in the post-period

Since the users may have reported symptoms with an increased prevalence in the post-period relative to the pre-period, we tried to estimate the duration of this behavioral change, hereafter referred to as symptom-reporting duration. To do so, we repeated pre-/post-comparisons for every symptom on modified timelines where weekly data from the post-period were iteratively removed until the increase of symptoms tweets was not significant. The procedure works as follows:

1. After removing tweets from week 0, we test whether the pre-/post-increase of symptom prevalence remains statistically significant for this modified dataset.
2. If the increase is not significant, that means that the symptom-reporting duration is shorter than 1 week and we consider the SRMD (i.e., symptom-reporting maximum duration) to be 1 week.
3. If the increase is significant, we additionally remove data from the next week and repeat the pre-/post-comparison.

This procedure can be applied iteratively to further weeks until the statistical test indicates no more significant changes between the pre-period and the modified post-period. Thus, each symptom can be associated with a specific SRMD that indicates the maximum number of weeks in the post-period accounting for the higher frequency of that particular symptom.

## 3. Results

### 3.1. Volume of test-positive tweets over time

**Figure 1:**
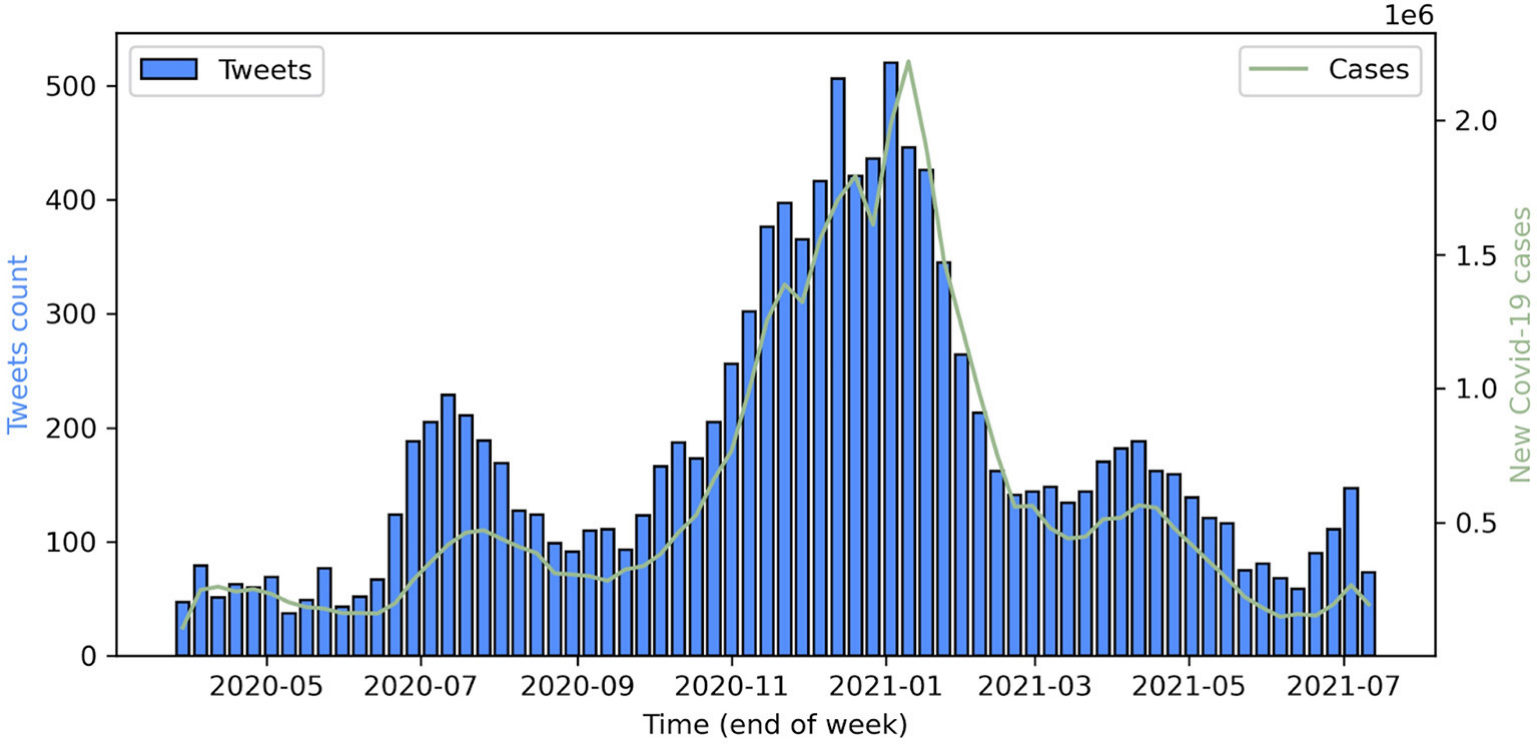
Blue bars: weekly counts of COVID-19 self-reports on Twitter data. Green line: cumulative confirmed new cases of COVID-19 in the USA, UK, Canada, and Australia.

Figure 1 shows the distribution of the primary test-positive tweets over the observation period of the study (March 2020 to July 2021) for all 12,121 users included in our dataset.

A local maximum of self-reported cases was observed in July 2020, which turns out to be the time at which a second wave of COVID-19 cases was observed in the United States. The bar plot reveals another maximum in late 2020 (November and December) and in early 2021 (January). This period coincides with the third wave of infections in the United States. Comparing the volume of case self-reports with the time series of cumulative confirmed new cases in the four largest countries where English is the main language (USA, UK, Canada, and Australia), we find a high correlation (Pearson's coefficient = 0.96, *p* < 1E-06), which is even stronger when delaying the tweets by a week (Pearson's coefficient = 0.97). A possible explanation is that people acknowledge their infection state with self-tests before the official PCR test results.

### 3.2. Emotions in test-positive tweets

After the emotions conveyed by the test-positive tweets were automatically identified with a state-of-the-art model (24), the possible classes (i.e., emotion labels) were ordered according to their frequency in this dataset. It turns out that there was no predominance of valence-negative emotions over valence-positive emotions (or vice-versa): for instance, the top four emotions were sadness, optimism, anticipation, and disgust (Supplementary Figure 2).

**Figure 2:**
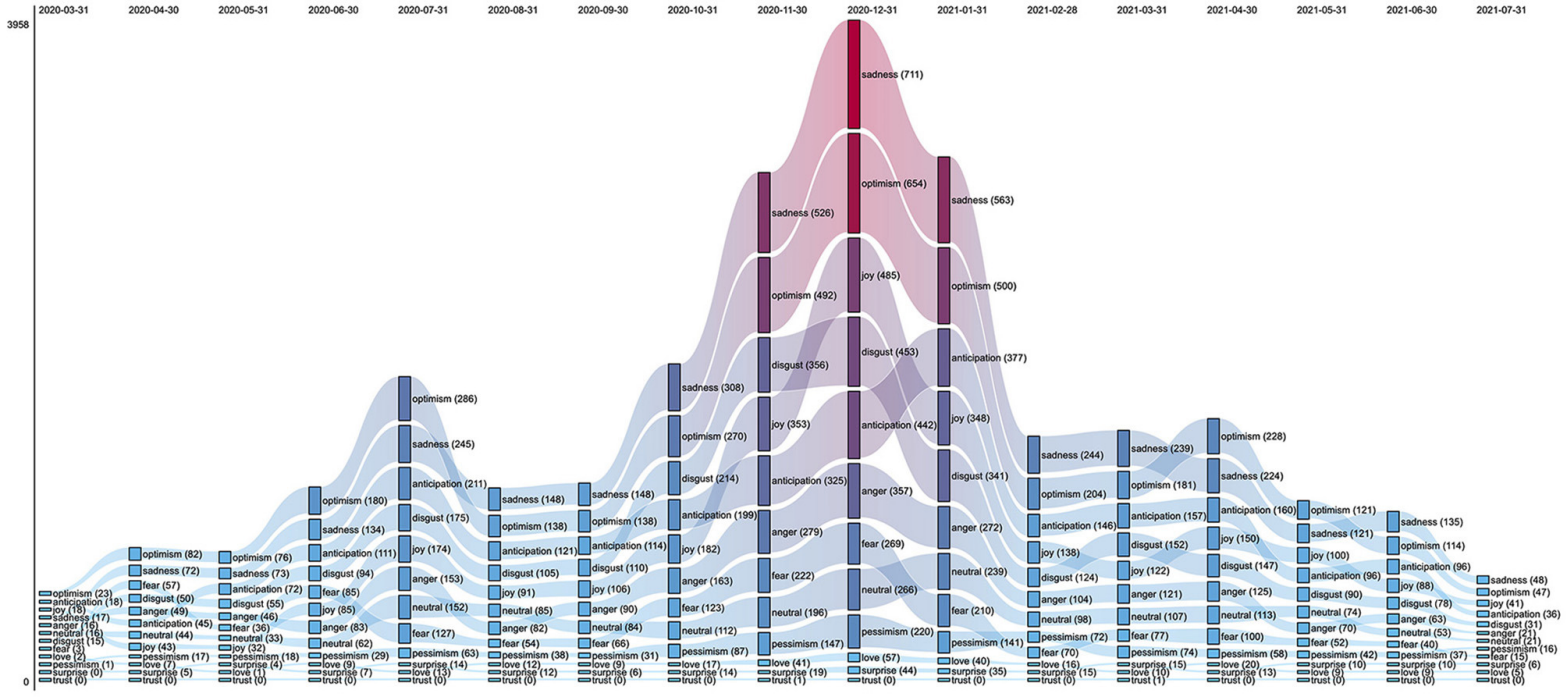
Ranked flow chart of the monthly counts of primary test-positive tweets, stratified by emotion. For each month, the emotions are sorted from the most abundant **(Top)** to the least abundant one **(Bottom)**.

Figure 2 illustrates the relative importance of emotions in the test-positive tweets based on their monthly prevalence and shows that sadness and optimism were the most frequent emotions across the entire time range (March 2020 to July 2021). While optimism was predominant until July 2020, it was superseded by sadness between August 2020 and March 2021. This change can be noticed when comparing the proportion of test-positive tweets with optimism or sadness in the first and second peaks (July 2020 and December 2020, respectively), as confirmed by a proportion test (*p*-value = 0.02).

The above flow chart also shows that ranking variations were relatively rare over time. Although this holds for most classes, two changes are worth noting. The relative importance of joy gradually increased from May 2020 (8th position) until December 2020 (3rd position) and later oscillated between the third and fifth rank (January 2021 to July 2021). Ranking fluctuations were also observed with respect to fear, which was the third and fifth most abundant emotion tag in April 2020 and June 2020, respectively, but then moved gradually to lower ranks until the end of the observation period.

### 3.3. Pre-/post-comparison

Each of the following subsections aims to characterize in greater detail each user's online activity before and after primary test-positive tweets were posted. As explained in the Methods section, the “pre-period” refers to the 3 months before the primary test-positive tweet of a user, while the “post-period” refers to the 3 months following the test-positive tweet.

#### 3.3.1. Volume of tweets

Using a causal impact analysis framework for each user separately, we compared the weekly series of tweets counts posted during the post-period with the corresponding series in the pre-period. Table 1 indicates that the tweets count time series were statistically not different in the pre- and post-periods for 42.1% of all the 12,121 users, while there was an increase for 34.8% of all users and a decrease for the remaining 23.1%.

**Table 1:** Relative size of the categories of users grouped according to the change of their individual activity on Twitter after the respective time of their primary test-positive tweet.

**Pre-/post-change**	**Number of users**	**Fraction of users [%]**
None	5,108	42.1
Increase	4,217	34.8
Decrease	2,796	23.1

This assessment is based on the results of causal impact analyses.

A second test was applied at the collective level: the same data were analyzed by applying a Wilcoxon signed-rank test on all the pre-/post-pairs (one pair per user), whose result indicates a statistically significant global increase in the weekly rate of tweets (*p*-value < 1E-06) during the post-period.

#### 3.3.2. Emotions

**Figure 3:**
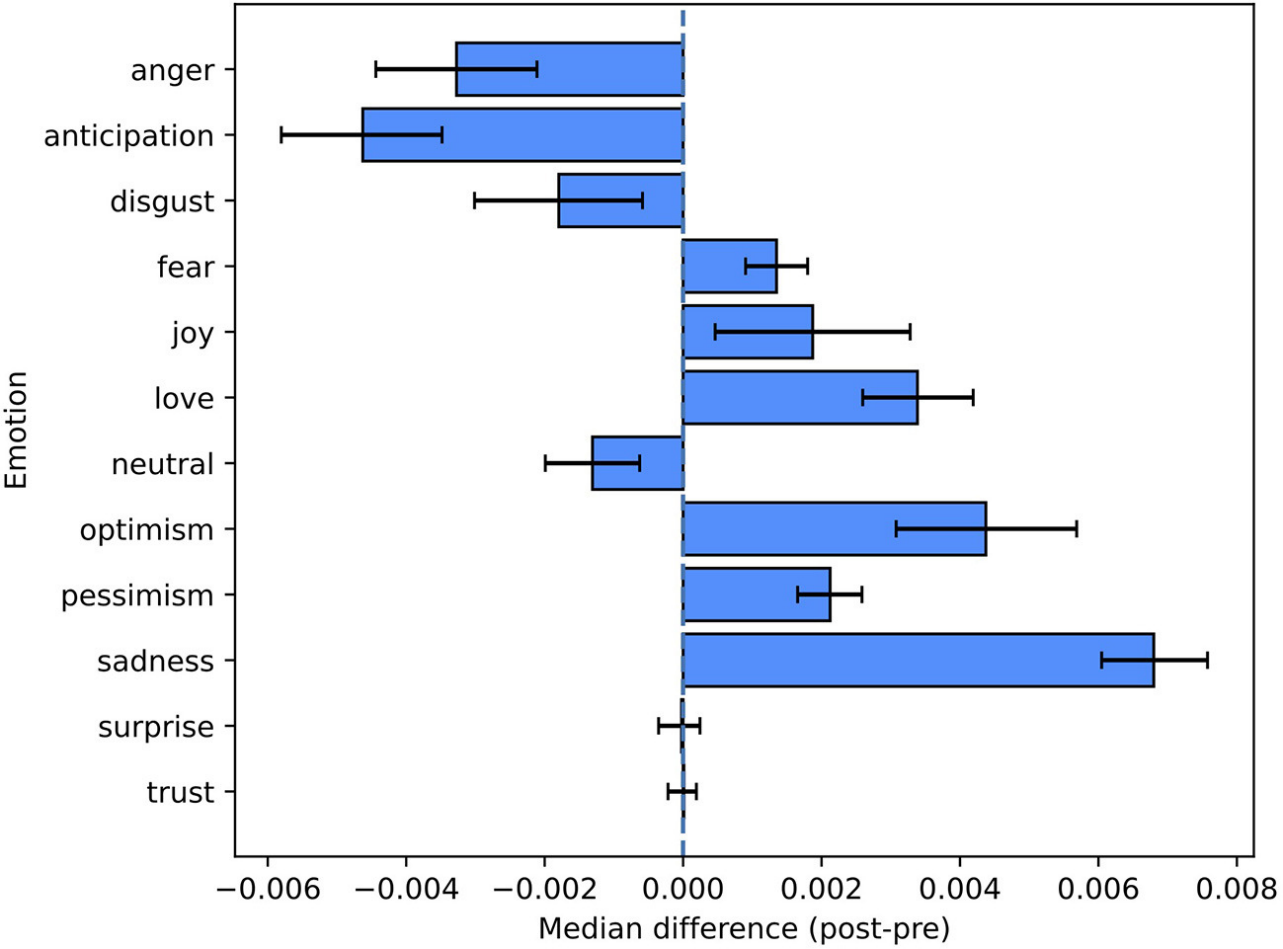
Pre-/post-comparison for emotion labels. This figure shows the non-parametric confidence intervals and the estimated pseudomedians of the differences between the proportions in the post-period and the ones in the pre-period.

Statistically significant changes between the pre-period and the post-period were observed for most emotions (see Figure 3 and Table 2).

**Table 2:** Emotion tags and pre-/post-comparisons.

**Emotion**	**Comparison**	**Adjusted *p*-value**	**Total number of occurrences**
Anger	Decrease	1E-07	1,542,110
Anticipation	Decrease	1E-14	1,529,341
Disgust	Decrease	9E-3	1,728,880
Fear	Increase	1E-08	233,686
Joy	Increase	2E-02	2,448,784
Love	Increase	3E-16	633,189
Neutrality	Decrease	4E-4	613,382
Optimism	Increase	1E-10	1,959,469
Pessimism	Increase	1E-18	242,033
Sadness	Increase	1E-68	628,846
Surprise	Unchanged	8.8E-01	94,519
Trust	Unchanged	9.8E-01	13,245

“Comparison” and “Adjusted p-value” correspond to the results of the multiple Wilcoxon signed-rank tests. The total number of occurrences is the number of tweets having the respective tag in the observation period of all the users.

Occurrences of anger, anticipation, and disgust were less frequent in the post-period, but there were also emotions (fear, joy, love, optimism, pessimism, sadness) displaying an opposite trend. The proportion of neutral tweets statistically decreased in the post-period (*p*-value = 2E-04).

#### 3.3.3. Topics

**Figure 4:**
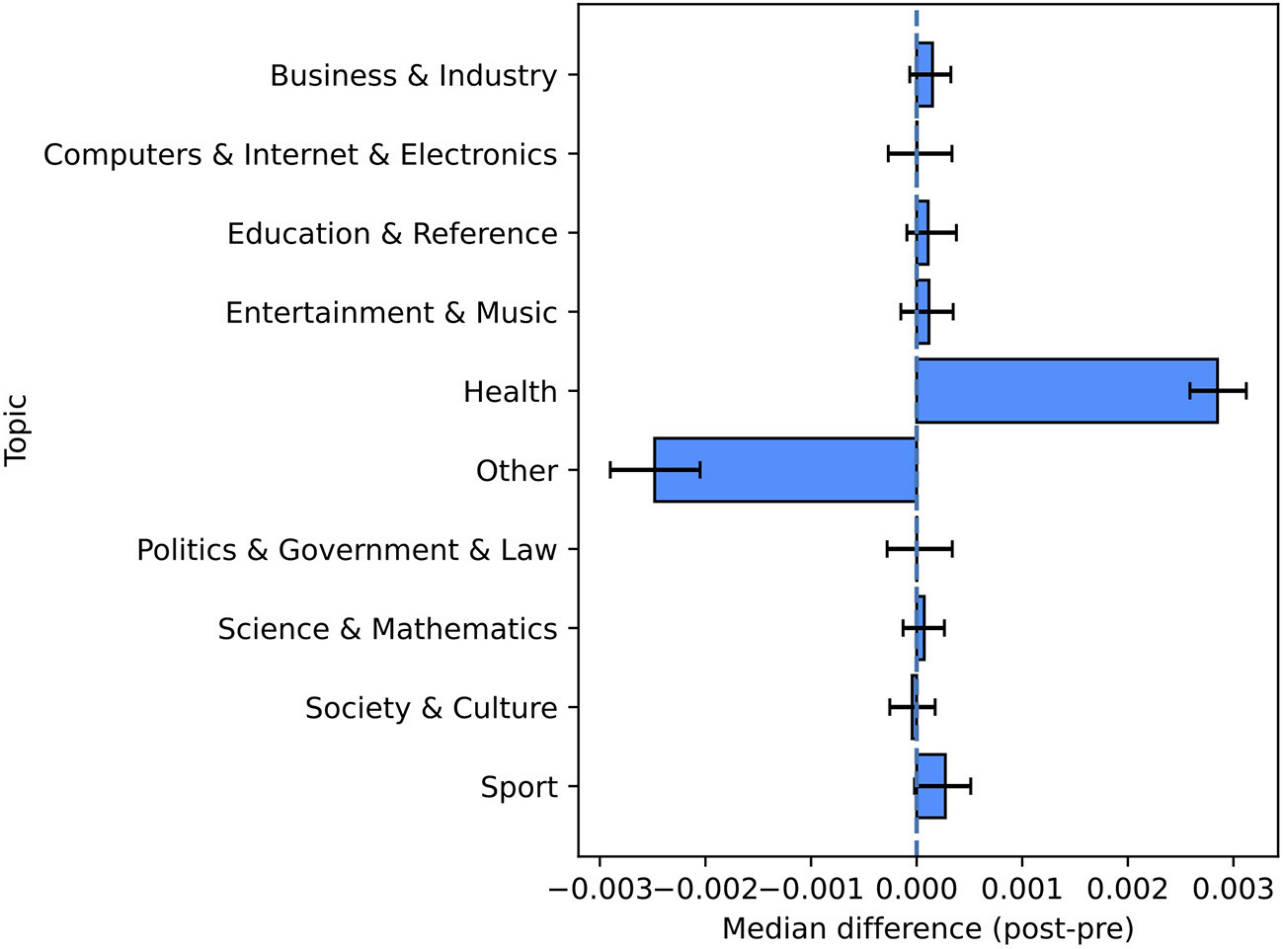
Pre-/post-comparison for the categories of topics. This figure shows the non-parametric confidence intervals and the estimated pseudomedians of the differences between the proportions in the post-period and the ones in the pre-period.

The above pre-/post-comparison shows that no statistically significant changes were observed for eight topic labels. The two remaining classes are the ”Other" category (less abundant after the primary test-positive tweets) and the “Health” category (more abundant in the post-period) (see Figure 4 and Table 3).

**Table 3:** Pre-/post-comparisons for the possible topics assigned to the tweets.

**Topic**	**Comparison**	**Adjusted *p*-value**	**Total number of occurrences**
Business and industry	Unchanged	3.0E-01	21,016
Computers and internet and electronics	Unchanged	9.1E-01	2,364
Education and reference	Unchanged	4.0E-01	4,429
Entertainment and music	Unchanged	6.0E-01	58,610
Health	Increase	3E-93	65,558
Other	Decrease	8E-28	5,678,023
Politics and government and law	Unchanged	9.3E-01	15,779
Science and mathematics	Unchanged	6.3E-01	4,052
Society and culture	Unchanged	8.8E-01	6,867
Sport	Unchanged	1.4E-01	91,853

The contents of the two central columns (“Comparison” and “Adjusted p-value”) correspond to the results of Wilcoxon signed-rank tests.

#### 3.3.4. Symptoms

**Figure 5:**
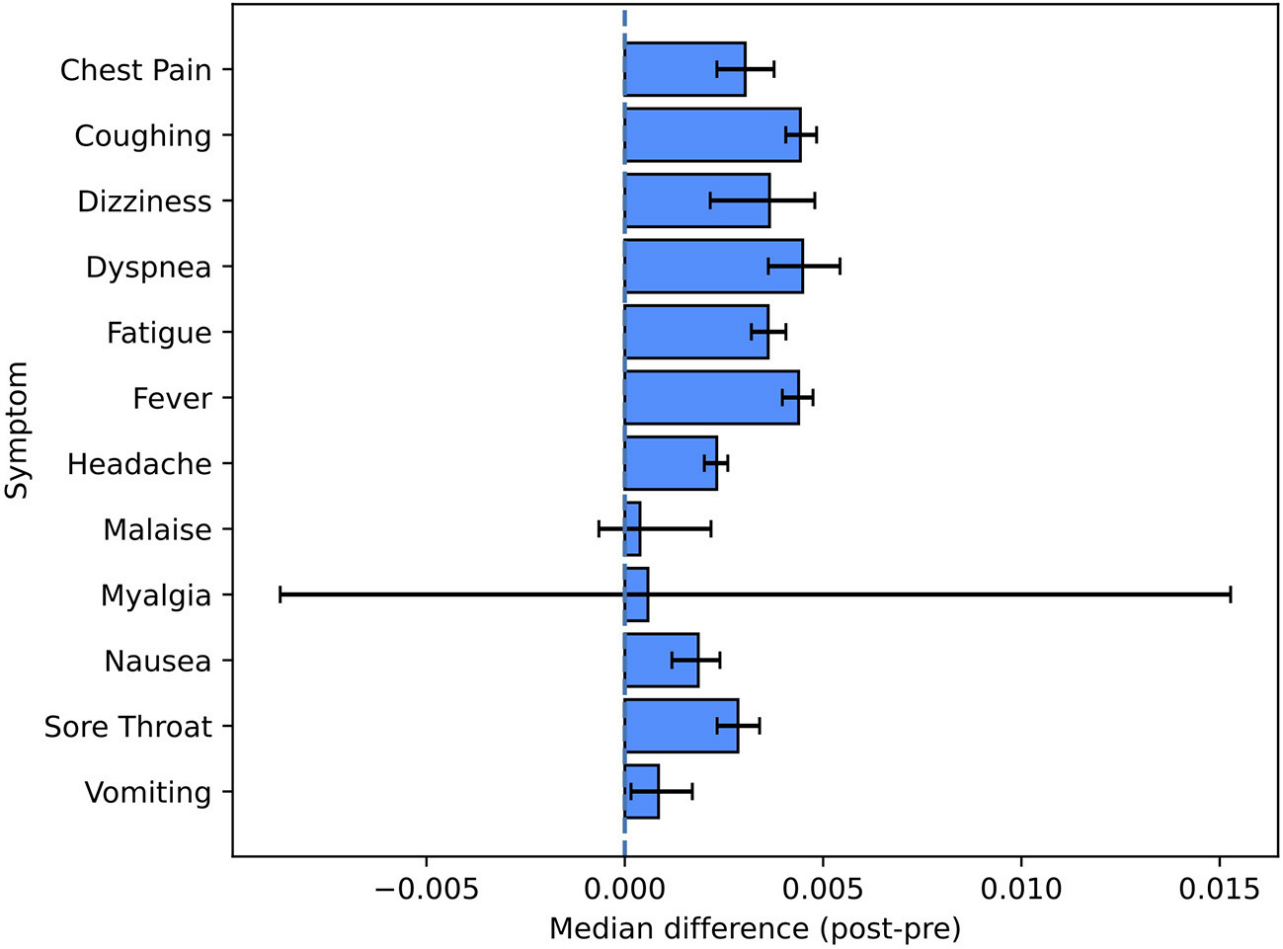
Pre-/post-comparison for symptom tags. This figure shows the non-parametric confidence intervals and the estimated pseudomedians of the differences between the proportions in the post-period and the ones in the pre-period.

Figure 5 and Table 4 report the changes observed for a set of 12 symptoms commonly found in people infected with COVID-19 (16). The most encountered symptoms in the post-period were headache, fever and coughing, which were present in 39.3%, 33.7% and 33.4% of the users reporting symptoms in the post-period, respectively. All the symptoms were more present in the post-period, with the exception of the least reported ones (myalgia and malaise) which had very low counts overall (12 and 38 occurrences for myalgia and malaise, respectively).

**Table 4:** Pre-/post-comparisons for the set of selected symptom tags.

**Symptom**	**Comparison**	**Adjusted *p*-value**	**Total number of occurrences**	**Percentage of users reporting the symptom in the post-period [%]**
Chest pain	Increase	2E-12	403	7.5
Coughing	Increase	1E-88	2,177	33.4
Dizziness	Increase	4E-4	99	1.9
Dyspnea	Increase	4E-18	296	6.2
Fatigue	Increase	6E-41	1,028	19.0
Fever	Increase	5E-93	2,262	33.7
Headache	Increase	2E-39	2,701	39.3
Malaise	Unchanged	6.9E-01	38	0.7
Myalgia	Unchanged	8.8E-01	12	0.1
Nausea	Increase	3E-08	806	12.3
Sore throat	Increase	1E-15	464	7.8
Vomiting	Increase	4E-02	298	4.9

The contents of the second and third columns (“Comparison,” “Adjusted p-value”) correspond to the results of the multiple Wilcoxon signed-rank tests.

Table 5 outlines the prevalence of the seven most frequent symptoms according to a medical study of COVID-19 cases reported to CDC between January 22, 2020 and May 30, 2020 (21). This table also provides the results of a study in which Twitter self-reports of symptoms were manually reviewed for a group of 203 users being infected by SARS-CoV-2 (study by Sarker et al.) (15). The post-period of our study covered 3 months (observation period of 12 weeks), while Sarker et al. considered all the tweets posted between February 1, 2020, and May 8, 2020.

**Table 5:** Comparison of the results obtained in the current study with two external studies: (i) the prevalence of symptoms in COVID-19 cases according to a medical study of cases reported to CDC between January 22, 2020 and May 30, 2020 (21); (ii) the results of a study based on manually reviewed Twitter data (15).

**Symptom**	**Symptom prevalence**	**Percentage of users reporting a given symptom in the post-period [%]**	**Percentage values in Sarker et al**.
Coughing	50.3	33.4	48.8
Fever	43.1	33.7	55.7
Myalgia	36.1	0.1	4.9
Headache	34.4	39.3	31.5
Dyspnea	28.5	6.2	30.5
Sore throat	20.0	7.8	20.2
Nausea	11.5	12.3	11.1

According to our data analysis, all twelve selected symptoms were most reported during the week of the test-positive tweets (see Supplementary Figure 3), which marked the beginning of the post-period. To determine whether the frequency increase in the post-period had a lasting effect, we estimated the symptom-reporting maximum duration (SRMD) for each symptom, as defined in the Methods.

Table 6 indicates that the higher prevalence of five symptoms (dizziness, headache, nausea, sore throat, and vomiting) in the post-period lasted 1 week. Five other symptoms (chest pain, coughing, dyspnea, fatigue, and fever) were characterized by a longer symptom-reporting duration (up to 4 weeks). In particular, the duration of the increase of dyspnea- and fatigue-related tweets lasts up to the 3rd and 4th week in the post-period, respectively. The symptom-reporting durations are partially in line with the duration of symptoms for mild SARS-CoV-2 infections, which has been estimated (29) as 31 ± 26 days for fatigue, 19 ± 22 days for dyspnea, 14 ± 13 days for coughing, 12 ± 6 days for nausea/vomiting, 11 ± 15 days for headache. On the other hand, the 2-week duration of fever reporting on Twitter is longer than the 6 ± 9 days duration of the symptom, and the 1-week duration of sore throat reporting is shorter than the 13 ± 17 days symptom.

**Table 6:** SRMDs of SARS-CoV-2 symptoms.

**Symptom**	**SRMD (weeks)**	***p*-value**
Chest pain	2	1.7E-01
Coughing	2	2.3E-01
Dizziness	1	1.8E-01
Dyspnea	3	1.1E-01
Fatigue	4	1.5E-01
Fever	2	2.1E-01
Headache	1	2.3E-01
Nausea	1	3.2E-01
Sore throat	1	7.0E-01
Vomiting	1	8.1E-01

An SRMD of n weeks means that the symptom-reporting duration is estimated to be ≤n weeks. The reported p-values are those of the Wilcoxon signed-rank tests which no longer indicated pre-/post-changes (see Section 2.8). E.g., The SRMD of Chest Pain is 2 weeks because the increase of Chest Pain reporting was not statistically significant when removing the first 2 weeks from the post-period.

## 4. Discussion

In this work we were interested in gaining insights into the content changes following self-reports of SARS-CoV-2 infection on Twitter. We used a large dataset of tweets posted by users who claimed to be infected between March 2020 and July 2021. Self-reports of infections were identified with an *ad-hoc* regular expression minimizing the presence of false positives, as we prioritized specificity over sensitivity. The correlation between the volume of self-reports detected on Twitter with the COVID-19 case numbers in the major English-speaking countries was high, which supports our assumption that the thus-identified tweets are indeed related to confirmed SARS-CoV-2 infections.

A recent study pointed out the predominance of negative emotions (anxiety, fear and sadness) on Twitter during the onset of the COVID-19 pandemic (30) with respect to a 2019 baseline (11). By applying a state-of-the art emotion classifier (24), we found that sadness was one of the two most represented emotions in self-reports of SARS-CoV-2 infections over the entire time range (March 2020 to July 2021). Nevertheless, our dataset also included a large proportion of tweets conveying optimistic content; this may reflect the effort of infected people to express positivity and hope through social media.

The prevalence of five symptoms (coughing, fever, myalgia, dyspnea, and sore throat) was underestimated in our results with respect to a CDC report (21), while the respective values for the prevalence of headache and nausea were consistent with this same report. In contrast, another study about self-reports of symptoms by Sarker et al. (15) reported percentage values (see rightmost column of Table 5) that are well aligned with the actual values of symptom prevalence found in the medical literature (21), confirming that Twitter can be valuable data source. Still, the self-reporting of symptoms in studies based on social media data is not systematic, which may affect the results. The gap between our results and these two studies (15, 21) points to a suboptimal sensitivity of our methodology, which may be a consequence of our attempt to maximize the specificity of symptom annotations. Finally, it is worth noting that both Sarker et al.'s study (15) and the CDC report (21) were produced during the early phase of the pandemic (between February 2020 and June 2020, that is, when most tested people were likely to have more severe symptoms as vaccines were not available yet), whereas our study was based on symptom reports from March 2020 up to October 2021. The symptom prevalence may have shifted over time due to changing testing strategies, the effects of the COVID-19 vaccination campaigns, and the evolving properties of the predominant SARS-CoV-2 strains.

Furthermore, our analysis shows that 10 out of the 12 analyzed symptoms were more represented in the post-period as compared to the pre-period. While half of the increases could be attributed to more frequent mentions in the very same week of the infection self-reports (dizziness, headache, nausea, vomiting, and sore throat), some others were longer-lasting (fatigue, dyspnea, chest pain, coughing, and fever). The number of weeks characterized by an increased symptom-reporting prevalence was in good agreement with the observed duration of clinical symptoms in COVID-19 mild cases (29). Even if we were not able to assess whether the collected tweets were actually related to SARS-CoV-2 infections, the significant increase of symptoms self-reports and the consistency with clinical symptom durations suggest that we captured relevant information related to confirmed infections. This approach can be used to automatically gain knowledge about symptomatology from Twitter data, to improve and inform traditional public health surveillance and studies.

One of the strengths of our study is that we retrieved the full public Twitter timeline for each user. This way, we were able to get an unbiased view on their behavior on Twitter, without the constraints that could have been induced by keywords-based tweets collections. Furthermore, we built a pre-infection baseline of tweets which allowed us to analyze the content changes of infected Twitter users. A key limitation of the study is that the data are entirely observational and derived from short-text messages, which may not grasp the full complexity of the symptoms. In addition, our method only finds a subset of users who were willing to voluntarily disclose their SARS-CoV-2 infection on Twitter, which may represent a biased sample. While the number of users sampled with our method is strongly correlated with official COVID-19 numbers, the results of the analysis may not be fully representative of the behavior before and after a SARS-CoV-2 infection in the general population. We do note, however, that the symptomatic observations from the sample are broadly in line with reported observations from clinical studies. Finally, while manually curated approaches seem to provide more accurate estimates of symptom prevalence, such methods are practically inapplicable to large volumes of data, but this tradeoff between data accuracy and volume will be reduced in the future as NLP methods improve.

Our study shows how social media can be used to aggregate cohorts of digital users on which to investigate the effects of infectious diseases. By comparing the content posted by Twitter users before and after self-reported SARS-CoV-2 infections, we observed an increase of emotional and health-related content. Additionally, we observed an increased proportion of symptom-containing tweets after SARS-CoV-2 infections were reported, and we found that the number of weeks accounting for the increase in symptom reports was in line with the duration of COVID-19 symptoms estimated in clinical studies. We therefore believe that in the same way that digital public health surveillance can be useful to detect acute health issues (31), the proposed approach may also be useful for slowly emerging long-term health issues such as post-acute sequelae of COVID-19. In particular, the full spectrum of symptoms could be searched for with a NER-based pipeline similar to ours, to identify all the possible symptoms associated to an infection. In general, digital health surveillance for novel phenomena that are slow to be recognized by the medical system (as was the case for post-acute sequelae of COVID-19) can be a useful complement to conventional health surveillance systems.

## Data availability statement

The code for the analyses described in this study can be found in the following GitHub repository: https://github.com/digitalepidemiologylab/content_changes_paper. Shareable data from this study can be found on Zenodo: https://doi.org/10.5281/zenodo.7499496.

## Author contributions

FD and FP collected and analyzed the data. FD, FP, DR, and MS designed the study and supervised the analysis. All authors wrote the paper. All authors contributed to the article and approved the submitted version.
