# Supplementary material for: Dynamics of social media behavior before and after SARS-CoV-2 infection
Source: Front Public Health. 2023 Feb 23;10:1069931. doi: 10.3389/fpubh.2022.1069931 (PMC9995964; doi:10.3389/fpubh.2022.1069931)
Supplement: Supplementary file 1 [file Data_Sheet_1.pdf]

Supplementary Material

1 Supplementary Data

1.1 Comparison between MedCAT and the lexicon of symptoms

Given the high agreement of Sarker et al. (15) with clinical data about symptom prevalence, we tested the lexicon of this study against the deep-learning MedCAT model. All tweets in the following analysis had to fulfill two conditions: (i) mentions of symptoms were identified in each of the tweets either by the MedCAT model or the lexicon-based approach; (ii) tweets were regarded as relevant for this analysis only if the probability for these tweets to be self-reports of symptoms was higher than 90% according to the deep learning model (23). Once these filtering steps were performed, a sample of 100 relevant tweets was selected. Two annotators (F.D. and F.P.) manually extracted the existing mentions of symptoms in each tweet. After the individual annotations were merged and a consensus was reached, the thus-obtained ground-truth labels were compared against the predictions obtained with MedCAT and the lexicon by using several metrics (precision, recall, specificity, F1 score). The corresponding results are provided in Supplementary Table 1.

We noticed that some phrases (“Pain”, “Sickness”, “Tired” and “Ache”) that are usually associated with symptoms are often used figuratively (e.g., “I am tired of hearing this song”). We therefore decided to exclude those symptoms from our analysis.

Then, our priority was to maximize the specificity (i.e., minimize the number of false positives) so as to make sure that the content changes observed in Results were associated with actual self-reports of symptoms. Supplementary Table 1 shows that MedCAT outperforms the lexicon-based approach according to the aforementioned metric. Given these results, we chose to apply MedCAT to tag symptom mentions in the full dataset.

1.2 URL classification

The dataset was also characterized by analyzing the text content hyperlinked from the URLs shared by the users in their tweets. These web pages were categorized with a method that combines a supervised classification algorithm and a database with a large number of categorized websites (32). Supplementary Table 2 shows that no significant rate changes were observed for many of the URL categories. Twitter users shared a smaller number of links to two types of Web pages (Arts and Entertainment, Law and Government) in the post-period.

2 Supplementary Figures and Tables

2.1 Supplementary Figures

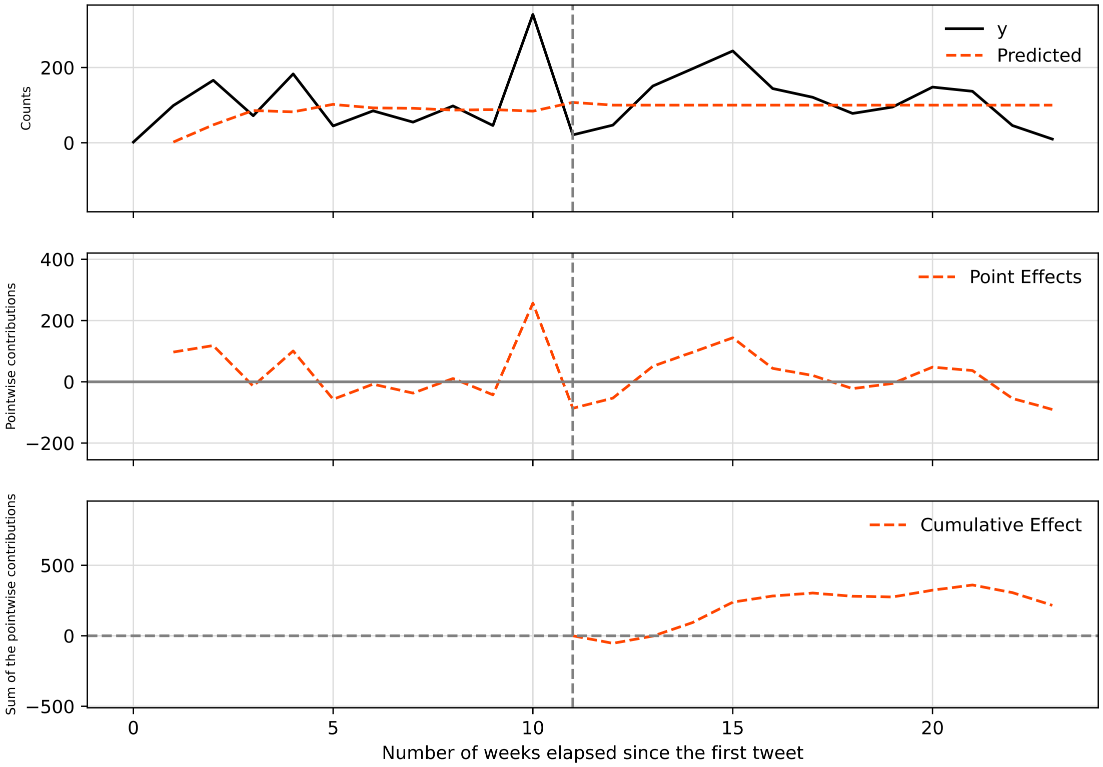

Supplementary Figure 1. Visualization of the results of an exemplified causal impact analysis with the Python's package *tfcausaIimpact* (27). The top panel represents the observed data points and the counterfactual time series in the post-period. The latter represents predictions generated with a Bayesian structural time series model (e.g., the default structural model used by the CausalImpact module is a so-called "local level", which is similar to a random walk). The central panel shows the pointwise causal effects, that is, the differences between the original and predicted time series. Finally, the bottom panel displays the cumulative effect of these differences, which corresponds to the summation of the pointwise effects over time.

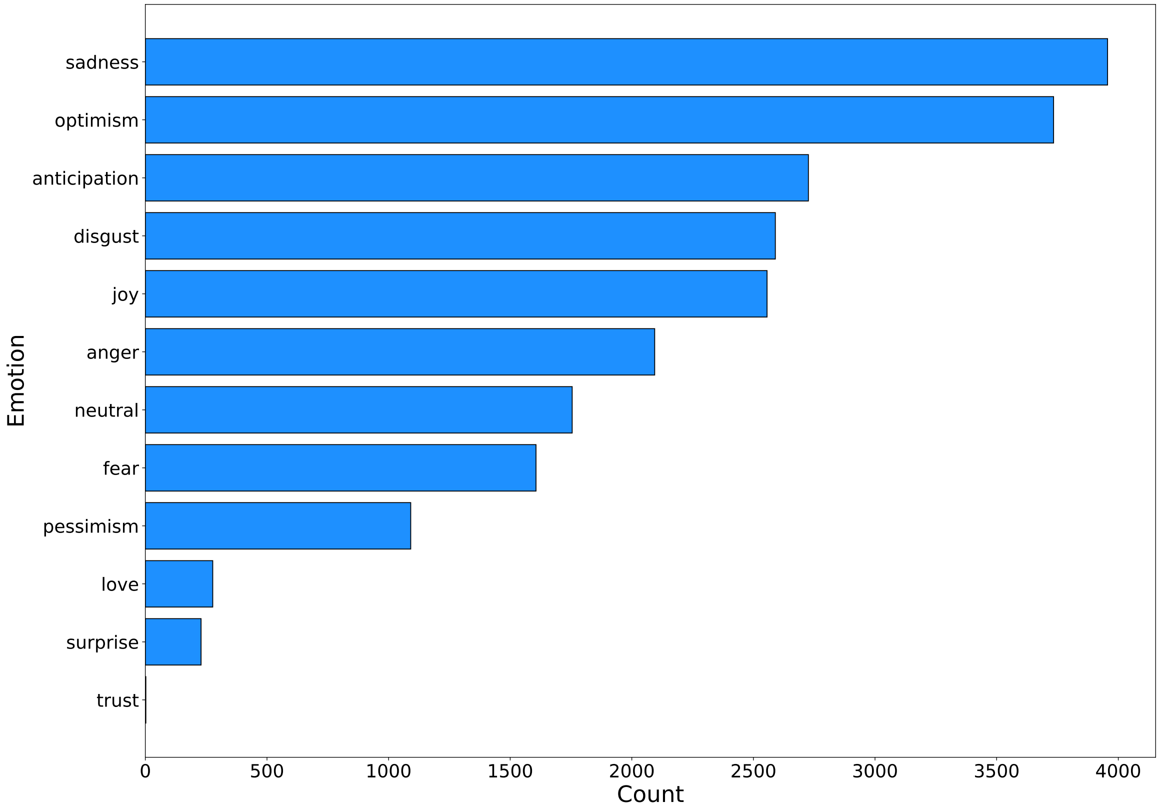

Supplementary Figure 2. Distribution of emotions in the tweets containing self-reports of COVID-19 infections. Emotion labels are ordered in descending order according to the counts identified in the test-positive tweets (i.e., tweets containing self-reports of COVID-19 infection). Sadness was the most present emotion label (3956 instances) followed by optimism (3734 instances), while trust was encountered only twice.

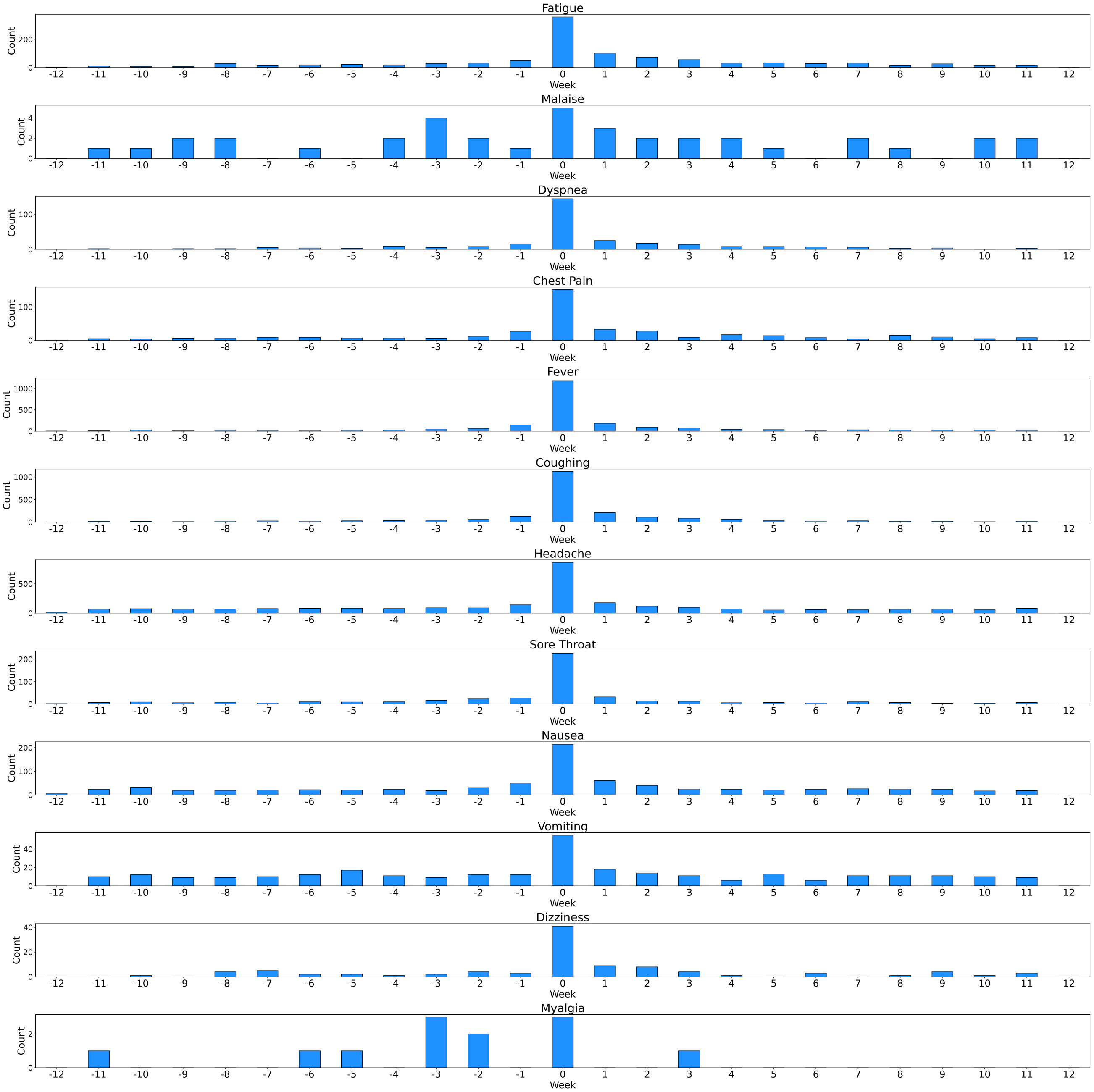

Supplementary Figure 3. Weekly counts (y-axis) of symptom mentions in the dataset. The x-axis refers to the week numbers on a relative timescale (pre-period → week number < 0; post-period → week number ≤ 0). Each of the twelve panels refers to a particular symptom commonly encountered in people having contracted COVID-19.

2.2 Supplementary Tables

Supplementary Table 1. Performance of MedCAT and the symptoms-lexicon on a random sample of 100 tweets from different users, annotated with at least one symptom by one of the two methods. Possibly ambiguous symptoms are “Pain”, “Sickness”, “Tired” and “Ache”.

	All symptoms (100 tweets)			
**Method**	**Precision**	**Recall**	**Specificity**	**F1 score**

MedCAT	5.7E-01	5.7E-01	1.7E-01	5.7E-01
Lexicon	6.7E-01	5.1E-01	4.1E-01	5.8E-01
	Not ambiguous symptoms (51 tweets)			
**Method**	**Precision**	**Recall**	**Specificity**	**F1 score**
MedCAT	6.9E-01	6.9E-01	4.2E-01	6.9E-01
Lexicon	6.8E-01	5.1E-01	2.6E-01	5.8E-01

Supplementary Table 2. Pre/post comparisons for the various URL categories. The results in the three central columns correspond to the results of the multiple Wilcoxon signed-rank tests.

Category	Comparison	Adjusted p value	Pseudomedian of the proportion difference (95% CI)	Total count
Adult	Unchanged	6.9E-01	1.83E-04 (-3.34E-04, 7.38E-04)	1,850
Arts and Entertainment	Decrease	6E-24	-1.31E-03 (-1.58E-03, -1.08E-03)	22,583
Autos and Vehicles	Unchanged	5.1E-01	-2.29E-04 (-7.02E-04, 2.44E-04)	3,243
Beauty and Fitness	Unchanged	8.8E-01	-7.31E-05 (-4.57E-04, 3.33E-04)	4,471
Books and Literature	Unchanged	9.8E-01	2.85E-05 (-3.79E-04, 4.00E-04)	5,950
Business and Industry	Unchanged	6.6E-01	-1.41E-04 (-6.40E-04, 2.91E-04)	1,754
Career and Education	Unchanged	3.9E-01	-3.60E-04 (-9.92E-04, 2.26E-04)	1,789
Computer and Electronics	Unchanged	4.7E-01	-1.88E-04 (-5.20E-04, 1.60E-04)	14,298
Finance	Unchanged	9.8E-01	2.06E-06 (-4.22E-04, 4.49E-04)	4,168
Food and Drink	Unchanged	9.1E-01	-3.67E-05 (-4.11E-04, 3.83E-04)	3,572
Gambling	Unchanged	9.0E-01	5.55E-05 (-4.43E-04, 5.45E-04)	9,031

Games	Unchanged	3.9E-01	-3.13E-04 (-8.04E-04, 1.93E-04)	36,744
Health	Unchanged	7E-01	4.79E-04 (1.40E-05, 9.31E-04)	14,396
Home and Garden	Unchanged	8.1E-01	2.01E-04 (-7.01E-04, 1.12E-03)	266
Internet and Telecom	Unchanged	7.1E-01	-1.23E-04 (-4.29E-04, 2.39E-04)	7,791
Law and Government	Decrease	2E-05	-9.72E-04 (-1.42E-03, -5.47E-04)	50,269
News and Media	Unchanged	6E-02	-3.24E-04 (-6.55E-04, -3.20E-05)	20,731
People and Society	Unchanged	3.6E-01	-2.65E-04 (-6.45E-04, 1.36E-04)	64,862
Pets and Animals	Unchanged	9.0E-01	5.59E-05 (-4.23E-04, 5.62E-04)	729
Recreation and Hobbies	Unchanged	6E-02	-5.31E-04 (-1.03E-03, -4.83E-05)	1,379
Reference	Unchanged	5.9E-01	-1.59E-04 (-5.59E-04, 2.36E-04)	5,291
Science	Unchanged	3.9E-01	-3.03E-04 (-7.85E-04, 1.71E-04)	6,618
Shopping	Unchanged	4.7E-01	2.42E-04 (-2.04E-04, 6.94E-04)	1,709
Sports	Unchanged	8.6E-01	-7.87E-05 (-4.44E-04, 2.61E-04)	10,229
Travel	Unchanged	2.0E-01	-5.07E-04 (-1.19E-03, 1.03E-04)	3,095

Supplementary Table 3. Numerical values associated with Figure 3. The pseudomedians of the proportion difference and the associated confidence intervals correspond to the results of the Wilcoxon signed-rank tests.

**Emotion**	**Comparison**	**Pseudomedian of the proportion difference (95% CI)**

Anger	Decrease	-3.27E-03 (-4.44E-03, -2.11E-03)
Anticipation	Decrease	-4.63E-03 (-5.80E-03, -3.48E-03)
Disgust	Decrease	-1.80E-03 (-3.01E-03, -5.86E-04)
Fear	Increase	1.35E-03 (9.03E-04, 1.80E-03)
Joy	Increase	1.87E-03 (4.63E-04, 3.28E-03)
Love	Increase	3.39E-03 (2.60E-03, 4.19E-03)
Neutrality	Decrease	-1.31E-03 (-1.99E-03, -6.28E-04)
Optimism	Increase	4.37E-03 (3.08E-03, 5.69E-03)
Pessimism	Increase	2.12E-03 (1.65E-03, 2.58E-03)
Sadness	Increase	6.80E-03 (6.05E-03, 7.58E-03)
Surprise	Unchanged	-2.27E-05 (-3.53E-04, 2.43E-04)
Trust	Unchanged	1.16E-05 (-2.17E-04, 1.92E-04)

Supplementary Table 4. Numerical values associated with Figure 4. The pseudomedians of the proportion difference and the associated confidence intervals correspond to the results of the Wilcoxon signed-rank tests.

**Topic**	**Comparison**	**Pseudomedian of the proportion difference (95% CI)**
Business & Industry	Unchanged	1.50E-04 (-6.52E-05, 3.24E-04)
Computers & Internet & Electronics	Unchanged	4.80E-06 (-2.69E-04, 3.34E-04)
Education & Reference	Unchanged	1.09E-04 (-9.21E-05, 3.77E-04)
Entertainment & Music	Unchanged	1.17E-04 (-1.50E-04, 3.46E-04)
Health	Increase	2.85E-03 (2.59E-03, 3.12E-03)
Other	Decrease	-2.48E-03 (-2.90E-03, -2.05E-03)
Politics & Government & Law	Unchanged	2.63E-06 (-2.79E-04, 3.37E-04)
Science & Mathematics	Unchanged	7.17E-05 (-1.28E-04, 2.63E-04)
Society & Culture	Unchanged	-4.27E-05 (-2.54E-04, 1.75E-04)
Sport	Unchanged	2.72E-04 (-2.03E-05, 5.12E-04)

Supplementary Table 5. Numerical values associated with Figure 5. The pseudomedians of the proportion difference and the associated confidence intervals correspond to the results of the Wilcoxon signed-rank tests.

Symptom	Comparison	Pseudomedian of the proportion difference (95% CI)
Chest Pain	Increase	3.04E-03 (2.32E-03, 3.76E-03)
Coughing	Increase	4.43E-03 (4.06E-03, 4.84E-03)
Dizziness	Increase	3.65E-03 (2.15E-03, 4.79E-03)
Dyspnea	Increase	4.49E-03 (3.62E-03, 5.43E-03)
Fatigue	Increase	3.61E-03 (3.19E-03, 4.06E-03)
Fever	Increase	4.38E-03 (3.97E-03, 4.74E-03)
Headache	Increase	2.32E-03 (2.01E-03, 2.60E-03)
Malaise	Unchanged	3.83E-04 (-6.52E-04, 2.17E-03)
Myalgia	Unchanged	5.83E-04 (-8.69E-03, 1.53E-02)
Nausea	Increase	1.85E-03 (1.19E-03, 2.40E-03)
Sore Throat	Increase	2.85E-03 (2.33E-03, 3.40E-03)
Vomiting	Increase	8.50E-04 (1.57E-04, 1.70E-03)

References

32. Meidus D. Website Classification Using Machine Learning Approaches [Internet]. Vilnius University; 2019 [cited 2022 Jul 15]. Available from: https://github.com/domantasm96/URL-categorization-using-machine-learning/blob/e4d49e6241168dcaf66b90fe6875a2c1b432bce6/Documentation/Website%20Classification%20Using%20Machine%20Learning%20Approaches.pdf
